# Medication Event Monitoring System for Infectious Tuberculosis Treatment in Morocco: A Retrospective Cohort Study

**DOI:** 10.3390/ijerph16030412

**Published:** 2019-01-31

**Authors:** Seup Park, Ilham Sentissi, Seung Jae Gil, Won-Seok Park, ByungKwon Oh, Ah Reum Son, Young Ju Kong, Sol Park, Eunseong Paek, Yong Joon Park, Seung Heon Lee

**Affiliations:** 1 Global Care International, Seoul 08377, Korea; park1993able@gmail.com (S.P.); hangye85@gmail.com (S.J.G.); wspark0222@gmail.com (W.-S.P.); rollzzang@gmail.com (B.O.); ariel.ahreum@gmail.com (A.R.S.); yj.kong@globalcare.or.kr (Y.J.K.); pes@globalcare.or.kr (E.P.); youngpark@gmail.com (Y.J.P.); 2 Chief Public Health Service and Epidemiological Surveillance, Moroccan League Against Tuberculosis (Ligue Marocaine de Lute Contre la Tuberculosis, LMCT), Rabat 10000, Morocco; sentissiiham@hotmail.fr; 3 Division of Pulmonary, Sleep and Critical Care Medicine, Department of Internal Medicine, Korea University Ansan Hospital, Ansan-City 15355, Korea; solpark@gmail.com

**Keywords:** tuberculosis, medication event monitoring system, success rate, lost to follow-up rate, Morocco

## Abstract

Non-adherence to tuberculosis (TB) treatment is a barrier to effective TB control. We investigated the effectiveness of a Medication Event Monitoring System (MEMS) as a tailored adherence-promoting intervention in Morocco. We compared patients who received a MEMS (n = 206) with patients who received standard TB care (n = 141) among new active TB patients with sputum smear-positive. The mean total medication days were 141.87 ± 29.5 in the control group and 140.85 ± 17.9 in the MEMS group (*p* = 0.7147), and the mean age and sex were not different between the two groups (*p* > 0.05). The treatment success rate was significantly higher in the MEMS group than in the control group (odds ratio (OR): 4.33, 95% confidence interval (CI): 2.13–8.81, *p* < 0.001), and the lost to follow-up rate was significantly lower in the MEMS group than in the control group (OR: 0.03, 95% CI: 0.05–0.24, *p* < 0.001) after adjusting for sex, age, and health centers. The mean drug adherence rate in the first month was significantly higher in the MEMS group than in the control group (*p* = 0.023). MEMS increased TB treatment success rate and decreased the lost to follow-up rate overall for infectious TB patients in a Moroccan rural area.

## 1. Introduction

Tuberculosis (TB) ranks as one of leading causes of death worldwide [1]. Standard treatment of tuberculosis can cure individual patients with drug-susceptible TB and minimize the transmission of *Mycobacterium tuberculosis* throughout the community [2]. Ensuring adherence to chemotherapy as a case management strategy is an important aspect alongside TB case finding in high TB burden countries [3]. Moreover, for patients undergoing TB treatment, education on treatment adherence is recommended by the World Health Organization (WHO), but treatment adherence interventions remain challenging [4].

Lack of adherence (lost to follow-up) to TB drugs increases the risk of treatment failure and drug resistance [5], and it has been reported that loss to follow-up is higher in older age groups than in younger age groups, in extra-pulmonary TB than in sputum smear-positive TB [6], and in re-treatment cases than in new treatment cases [7]. In addition, loss to follow-up amongst adverse treatment outcomes all occurred within the first two months of treatment initiation. For infectious TB diseases that require lengthy treatment periods, an effective monitoring system to prevent treatment interruption is essential to prevent the uncontrolled spread of infectious disease throughout the community [8].

With the introduction of Directly Observed Treatment Short Course (DOTS) in 1991, Morocco saw a significant drop in the incidence and mortality rate of tuberculosis [9]. However, the TB incidence rate remained rather high, with 103 cases per 100,000 population in 2014. It was even higher in populated cities like Sale and Temara (197 cases per 100,000 population), and the overall treatment success rate seemed to be as low as 84% with a lost to follow-up (non-compliance) rate as high as 15%, according to the WHO [10]. With limited human resources, as is the case in Morocco, the conventional face-to-face approach for effective TB control had limited success [10]. Therefore, real time awareness programs, such as mobile health (m-health) solutions, can be an alternative method for improving drug adherence to disease treatment as they allow the healthcare professional to communicate with and monitor patients from a distance by shortening communication pathways [11].

Mobile technology has been used in other diagnostic fields, image transmission for telecommunication, case finding strategies for TB in hard-to-reach areas, adherence monitoring, and visit reminders [12] using short message service (SMS) feedback and video-observed therapy (VOT) [13,14]. Effective pillbox intervention was reported in chronic diseases such as hypertension [15]. Furthermore, pillboxes linked to a web-based application have been introduced to improve treatment adherence in TB patients. These surpassed both the labor-intensive DOT strategy [16] as well as standard TB care [17]. Recently, the WHO endorsed the use of Digital DOTS as a replacement for traditional DOTS [4], but the effectiveness of digital health strategies still requires further research. We have launched a smart pillbox (SPs) with e-promms [14,16], namely the Medication Event Monitoring System (MEMS) as a tailored adherence-promoting intervention for improving patients’ adherence to TB treatment in urban areas of Morocco.

The aim of this paper is to investigate the effectiveness of the MEMS program by analyzing and comparing the initial data of the MEMS program with the conventional TB control program. 

## 2. Materials and Methods

### 2.1. Morocco Guidelines for Tuberculosis Treatment

The national TB treatment guidelines of Morocco recommend two months of rifampin, isoniazid, pyrazinamide, and Ethambutol (2RHZE) (six days out of seven for two months, a total of 56 days) followed by four months of rifampicin and isoniazid (4RH) (six out of seven days for four months, a total of 112 days) for new smear-positive cases as a category I treatment regimen [18,19]. Retreatment regimens include two months of streptomycin, rifampin, isoniazid, pyrazinamide, and Ethambutol (SRHZE), followed by one month of RHZE and five months of RHE (2SRHZE/1RHZE/5RHE). Direct supervision at the treatment site is recommended by Moroccan guidelines before self-administered treatment (SAT) [19]. For new pulmonary TB with sputum acid fast bacilli (AFB) positive cases, sputum AFB smear follow-up is recommended at the second month of treatment, and the effectiveness of treatment is measured both clinically and bacteriologically.

### 2.2. Smart Pillbox and Medication Event Monitoring System with E-Promms

The smart pillbox (SPs) as a digital health technology is a medication storage device [14,16] that sends data in real-time via a subscriber identity module (SIM) card to a web-based application based on a telecommunication interface (Wi-Fi, 3G/4G, Ethernet). Basically, SPs contain a load cell sensor which can measure the weight of a medicine with a resolution of 0.01 g. E-promms, a web-based medication monitoring system, is a central part of the MEMS that can be accessed at any time by health care workers. It provides periodic statistical monitoring results including drug adherence status according to time, located region, and patient information. It integrates the information, and it sends notifications to the SPs to remind patients to take medication at certain times. When patients fail to take their scheduled medication, health care workers promptly respond and take appropriate actions such as phone calls or home visits. Details of the data transmitted between SPs and MEMS are as follows: (1) from SPs to MEMS: patient identification (ID), SPs ID, changed weight of medicine, and SPs’ door opening and closing. (2) from MEMS to SPs: patient ID, SPs ID, alarm time, multimedia message for patients, and remote-control command for SPs. We adopted an health level 7 versions (HL7 v2.5) communication protocol, which is a set of international standards used by various health care providers [20]. For security, a secure sockets layer (SSL) protocol was used [21]. For privacy protection, user authentication and hierarchical user authorization were adopted for access to MEMS in addition to obtaining initial agreement from the patients.

### 2.3. Operation Oversight and Study Population

The pilot operation, which had been implemented in Sale, Temara, Casablanca, and Khemisset as a national TB control program under the control of the local health ministry of Morocco from April 2014 to March 2015, was reviewed. The 347 new sputum smear-positive TB patients were reviewed retrospectively in order to identify the effectiveness of the MEMS. The number of patients in the intervention group and control group were 206 and 141, respectively. The patients who had wanted and agreed to use SPs without randomization were regarded as the intervention group

New sputum smear-positive (SSM+) TB patients were provided with SPs. The SP intervention was initialized at five health centers (Bab Khmiss, Sidi Mousa, Hay Rahma, Laayayda, and Hay Salam II) in the Sale area of the Rabat–Sale–Kenitra region. Information from SPs was sent to e-promms via a 3G modem in real time. Patients in the control group were selected from newly diagnosed TB patients confirmed by a sputum AFB test and registered in the rest of the health centers in the Sale area (Bab Sebta, Bettana, Bouknadal, Douar Cheikh Imfadal, Jarda, Pepiniere, and Tabriquet). MEMS in the intervention group were applied after one to two weeks of routine direct supervision for medication at the treatment site, depending on the specific situation. Routine standard treatment composed of direct supervision for the initial one to two weeks and self-administered treatment (SAT) for the remaining periods was performed on the control group. Pilled blister packs for fixed drug combinations were inspected by nurses to monitor compliance to treatment when the patient came to a health center regularly to receive additional drugs. Other treatment monitoring, including sputum AFB smears, followed the national guidelines for TB in Morocco [16]. The study protocol was approved by the Institutional Review Board (IRB) of Mohammed V University (IRB number: dossier number 48/16).

### 2.4. Definition of Treatment Outcomes

The definition of treatment outcomes was based on 2013 WHO revised definitions [22]: (1) cured—a pulmonary TB patient with bacteriologically confirmed TB at the beginning of treatment who was smear or culture-negative in the last month of treatment and on at least one previous occasion; and (2) treatment completed—a TB patient who completed treatment without evidence of failure, but with no record to show that sputum smear or culture results in the last month of treatment and on at least one previous occasion were negative, either because tests were not done or because results were unavailable. Treatment success was the sum of cured and treatment completed. Lost to follow-up was defined as a TB patient who initiated TB medication but was lost to follow-up.

### 2.5. Statistical Analysis

All analyses were performed using SPSS software, version 20.0 (SPSS Inc., Chicago, IL, USA). *t*-test, ≅χ^2^ test, Fisher’s exact test, and a logistic regression model were used to determine differences between the MEMS group and the control group. A mixed-effect model was used to estimate the longitudinal course of accumulated drug adherence rate for the MEMS group and control group. According to log-likelihood values, the best fit for the correlation structure was determined to be a compound symmetry structure, and random effects were applied to the slope as well as to the intercept. In addition, analysis of variance (ANOVA) was used in order to determine differences between the two groups based on each monthly accumulated drug adherence rate. Statistical significance was set at *p* < 0.05.

## 3. Results

### 3.1. Clinical Characteristics of the Participants

A total of 347 participants were analyzed in this study, as shown in Table 1, after excluding 15 patients. The 15 excluded patients, from several different health centers, initially agreed to pillbox (MEMS) usage, but the digital box system could not be set up in their homes due to private issues (crime: 1, divorce: 2, house moving: 2, home repair: 2, conflicts between family members: 5, unrevealed issues: 3). These patients were converted to routine standard treatment and excluded from this analysis. The baseline characteristics of the participants are shown in Table 1, and categorized by the control group (n = 141) and MEMS group (n = 206). All participants were newly diagnosed with active pulmonary TB confirmed by a positive sputum AFB result. The mean age was not different between the two groups (35.7 ± 16.3 years and 36.7 ± 14.9 years, *p* = 0.577). The groups had 68.8% and 71.4% males, respectively, with no statistical difference (*p* = 0.693). The mean total medication days were 141.87 ± 29.5 days in the control group and 140.85 ± 17.9 days in the MEMS group, respectively (*p* = 0.7147). The mean drug adherence rate was 80.77 ± 9.2% and 81.35 ± 6.8%, respectively, without statistical significance (*p* = 0.525). However, based on the treatment results, the cure rate was significantly higher in the MEMS group (62.1%) than in the control group (43.3%) (*p* = 0.001), and the lost to follow-up rate was significantly lower in the MEMS group (0.5%) than in the control group (10.6%) (*p* < 0.001). As a result, the treatment success rate was significantly higher in the MEMS group (93.2%) than in the control group (79.5%) (*p* < 0.001).

### 3.2. Comparison of Treatment Results between the Medication Event Monitoring System Group and Control Group

With logistic regression analysis after adjusting for sex, age, and health centers, as shown in Table 2, treatment success was shown to be significantly higher in the MEMS group (odds ratio (OR): 4.33, 95% confidence interval (CI): 2.13–8.81, *p* < 0.001) compared to the control group, and lost to follow-up was significantly lower in the MEMS group than in the control group (OR: 0.03, 95% CI: 0.05–0.24, *p* = 0.001). The health center was irrelevant to lost to follow-up (OR: 1.11, 95% CI: 0.97–1.27, *p* = 0.128), but men had a significantly higher lost to follow-up rate than women (OR: 8.58, 95% CI: 1.07–68.45, *p* = 0.043).

### 3.3. Time Series Analysis of the Efficacy of Medication Event Monitoring System

**Figure 1:**
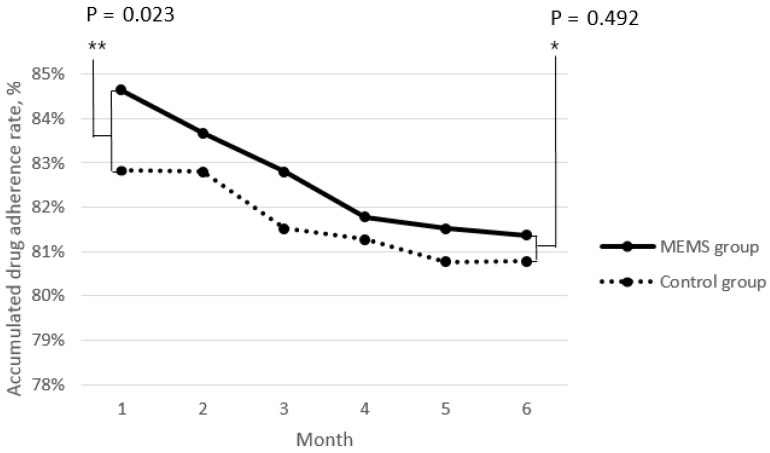
Accumulated drug adherence rate: The mean drug adherence rate in the first month (**) was significantly higher in the MEMS group than in the control group, but not in the final sixth month (*).

Outcomes from the linear mixed-effects models are presented in Table 3. The seclining slopes for the accumulated drug adherence rate ranged from as steep as −0.62% (*p* < 0.001) per additional month of treatment with statistical significance, but from −0.02% (*p* = 0.077) per additional year of age without statistical significance. The accumulated drug adherence rate over time was significantly higher in the male group than in the female group (drug adherence rate estimate = 0.86, 95% CI: 0.18–1.55, *p* = 0.014). After adjusting these covariates, the MEMS group showed a significantly higher accumulated drug adherence rate than the control group (*p* = 0.002). As shown in Figure 1, which represents the accumulated drug adherence rate for six months of treatment, the mean drug adherence rate in the first month was significantly higher in the MEMS group than in the control group (*p* = 0.023), but there were no differences between the two groups in the remaining five months including the final sixth month (*p* = 0.492).

## 4. Discussion

Medication Event Monitoring System improved the TB treatment success rates and decreased the abrupt follow-up loss for infectious TB patients in this study. Over time, the MEMS group showed a higher monthly accumulated drug adherence rate compared to the standard conventional TB treatment over a six-month period. MEMS is expected to contribute to the effectiveness of the TB case management strategy based on its convenient and effective monitoring of medication in rural Moroccan areas, where the TB burden is high.

There have only been a few reports about electronic TB treatment adherence devices, most with a small number of enrolled patients [14]. This study, however, targeted infectious TB patients with sputum AFB smear-positive with enough control subjects and investigated the effect over time. Medication Event Monitoring System seemed to be very beneficial for controlling TB infectiousness given that it enhanced treatment compliance during the first two months of the intensive treatment period when the negative conversion of sputum AFB culture develops in drug-susceptible TB [2].

Based on the time series analysis, as in other research papers, the accumulated drug adherence rate decreased over time [23]. In our study, the lost to follow-up rate was significantly higher in men than in women, similar to a previous report [18]. However, women had a tendency to skip medications intermittently, but to finish out the terms of medications for 6 months, which was potentially related to the circumstances of certain Islamic cultures, where female patients are comparatively not as free to access public health centers as men [24]. For the patients who were lost to follow-up, the information in e-promms linked to Google Maps and geographical pictures was useful for health care workers to visit patients’ homes, and the geographic information system (GIS) could to be applied to MEMS to manage patients lost to follow-up and contact with infectious TB efficiently [25]. However, the MEMS group consistently showed a higher accumulated drug adherence rate than the control group throughout the six months of treatment because of the pronounced gap between the two groups in the first month of treatment (*p* = 0.029). Therefore, MEMS is expected to have a higher impact on the initial period of TB treatment for infectious TB patients in terms of encouraging them to continue with the medication.

The national Moroccan guidelines recommend standard DOT during the initial two months of treatment for new severe cases, relapsed or failed cases, and multi-drug resistant (MDR) cases [19]. Because far greater commitment is needed in the setting of drug-resistant TB than with the drug-susceptible disease [26] in the context of higher non-adherence with high pill-burden drugs [27], this system is expected to be useful in other categories of treatment, such as drug-resistant cases, and is currently being investigated in other sites in Morocco.

There were some limitations to our study. First, as this study was retrospective, it was difficult to identify other clinical information including consecutive AFB culture results, combined comorbid conditions of patients, adverse drug events, and socioeconomic status. Therefore, treatment outcomes relying upon the AFB result could not be reconfirmed if the reported treatment outcome was “cured” or “treatment completed” based on the AFB result. Second, besides counting pilled blister packs regularly, no diagnostic method such as urine stick was used to confirm that patients had really taken their medication. Third, there could have been a Hawthorne effect, where better treatment outcomes in the MEMS group could be due to the patients’ awareness of being observed [28]. Last, the development of a well-structured m-health system, cooperation with local health workers, and human resource development seemed to be vital for successful and relevant data acquisition toward deeper investigations in a future. In this study, the per-patient total cost covering equipment, training, overhead, and direct human resources to complete TB treatment using MEMS was $321, but a more detailed cost–utility analysis that evaluates all the investment of human resources based on estimates of the cost per disability adjusted life years (DALY) is essential to implement MEMS in an actual national TB control program (NTP). Moreover, further study on a larger scale with new technologies equipped with video and GIS systems for latent TB and for TB contacts as well as for drug-resistant TB should be performed to demonstrate the usefulness of this mobile health technology in the field of infectious TB control.

## 5. Conclusions

In conclusion, MEMS based on an m-health-based TB monitoring program played a successful role in reducing the number of lost to follow-up infectious TB patients, especially during the intensive phase, as well as improving the treatment success rate in a rural area in Morocco with high TB-prevalence. However, a large prospective study including a cost-effectiveness study is warranted, and keen attention and help from the NTP is vital to validate and launch a digital DOT system in Morocco.

## Figures and Tables

**Table 1:** Comparison of general characteristics and treatment results between the control group and the Medication Event Monitoring System (MEMS) group.

	Controls (n = 141)	MEMS Group (n = 206)	*p* Value
**Age (mean, SD)**	35.74 (16.3)	36.70 (14.9)	0.5772
**Male gender (n, %)**	97 (68.8%)	147 (71.4%)	0.6936
**Patients with sputum AFB smear +ve (n, %)**	141 (100%)	206 (100%)	
**Treatment type**			
New case, category I (n, %)	141 (100%)	206 (100%)	0.488
**Total follow-up days (mean, SD)**	175.2 (29.4)	173.4 (18.7)	0.488
**Total medication days, (mean, SD)**	141.87 (29.5)	140.85 (17.9)	0.7147
**Drug adherence rate, (mean, SD)**	80.77 (9.2)	81.35 (6.8)	0.525
**Numbers of patients who belong to treatment success (n, %)**	112 (79.5%)	191 (93.2%)	<0.001
**Treatment outcome**			
Cured (n, %)	61 (43.3%)	128 (62.1%)	0.001
Treatment completed (n, %)	51 (36.2%)	64 (31.1%)	0.321
Treatment failed (n, %)	3 (2.1%)	0 (0.0%)	0.066
Died (n, %)	2 (1.4%)	1 (0.5%)	0.569
Lost to follow-up (n, %)	15 (10.6%)	1 (0.5%)	<0.001
Not evaluated (n, %)	9 (6.4%)	12 (5.8%)	0.831
Treatment success (n, %)	112 (79.5%)	192 (93.2%)	<0.001

Abbreviations: SD, standard deviation; AFB, acid fast bacilli. +ve = positive.

**Table 2:** Logistic regression model assessing independent predictors of treatment success and lost to follow-up for the case holding strategy for tuberculosis (TB) treatment.

Variable		Odds Ratio	95% CI	*p* Value
Treatment success	MEMS	4.33	2.13–8.81	<0.001
	Controls	Ref.		
	Sex, Male	0.49	0.22–1.10	0.085
	Age	0.99	0.98–1.02	0.657
	Health centers	0.90	0.83–0.98	0.012
Lost to follow-up	MEMS	0.03	0.05–0.24	0.001
	Controls	Ref.		
	Sex, Male	8.58	1.07–68.45	0.043
	Age	1.01	0.97–1.04	0.825
	Health Centers	1.11	0.97–1.27	0.128

Abbreviations: MEMS, Medication Event Monitoring System; Ref., reference.

**Table 3:** Generalized linear mixed model estimating the time course of accumulated monthly drug adherence rate for TB medication over six months.

Variable	Drug Adherence Rate (%) Estimate	95% CI	*p* Value
MEMS	1.02	0.40–1.66	0.002
Controls	Ref.		
Age	−0.02	−0.04–0.002	0.077
Time, Month	−0.62	−0.80–−0.44	<0.001
Gender (Male)	0.86	0.18–1.55	0.014

Abbreviations: MEMS, Medication Event Monitoring System; CI, confidence interval; Ref., reference.
